# Diagnosis and treatment of cystic renal cell carcinoma

**DOI:** 10.1186/1477-7819-11-158

**Published:** 2013-07-17

**Authors:** Jiexiu Zhang, Bianjiang Liu, Ninghong Song, Lixin Hua, Zengjun Wang, Min Gu, Changjun Yin

**Affiliations:** 1 Department of Urology, The First Affiliated Hospital of Nanjing Medical University, Nanjing, 210029, China

**Keywords:** Cystic renal cell carcinoma, Diagnosis, Nephrectomy, Nephron-sparing surgery

## Abstract

**Background:**

To summarize the diagnosis and treatment of cystic renal cell carcinoma (CRCC).

**Methods:**

A retrospective study was conducted on 13 patients with CRCC at our center from August 2004 to April 2012. The pathologic features, clinical manifestation, imaging characteristics, treatment, and prognosis of CRCC were summarized according to available literature.

**Results:**

Of the 13 patients, 11 were diagnosed with CRCC by preoperative B ultrasonography and computed tomography (CT) scan. The remaining two cases were initially misdiagnosed with simple renal cysts. Open radical nephrectomy was performed on two of the 13 cases, laparoscopic radical nephrectomy on seven cases, and open partial nephrectomy on four cases. All diagnoses of CRCC were confirmed by pathological examination. After the operation, all patients had an uneventful recovery. During the follow-up (range, 6–60 months), the serum creatinine concentrations and GFR of the partially removed kidneys remained stable within the normal range. No tumor recurrence or metastasis occurred.

**Conclusions:**

By combining imaging examinations (B ultrasonography and CT scan) with intraoperative pathological examination, most cases of CRCC can be diagnosed and treated promptly and accurately. Nephrectomy is the first-line therapy. Nephron-sparing surgery should be preferred for CRCC. After a successful operation, the prognosis of CRCC is good.

## Background

Cystic renal cell carcinoma (CRCC) is a special type of renal cell carcinoma. It is relatively rare and involves fluid-filled masses. The classification of cystic renal disease is based on the Bosniak classification system (Table 1) [1,2]. However, CRCC is usually misdiagnosed as a benign renal cyst due to similar clinical manifestations and imaging characteristics. In the present study, we retrospectively analyzed 13 cases with CRCC at our center and summarize the pathologic features, clinical manifestation, imaging characteristics, treatment, and prognosis of CRCC according to available literature.

**Table 1:** The Bosniak classification of renal cystic masses

**Bosniak category**	**Features**	**Work**-**up**
I	A simple benign cyst with a hairline-thin wall that does not contain septa, calcification, or solid components. It has the same density as water and does not enhance with contrast medium.	Benign
II	A benign cyst that may contain a few hairline-thin septa. Fine calcification may be present in the wall or septa. Uniformly high-attenuation lesions <3 cm in size, with sharp margins but without enhancement.	Benign
IIF	These cysts may contain more hairline-thin septa. Minimal enhancement of a hairline-thin septum or wall can be seen. There may be minimal thickening of the septa or wall. The cyst may contain calcification, which may be nodular and thick, but there is no contrast enhancement. There are no enhancing soft-tissue elements. This category also includes totally intrarenal, non-enhancing, highattenuation renal lesions ≥3 cm in size. These lesions are generally well-marginated.	Follow-up. A small proportion are malignant.
III	These lesions are indeterminate cystic masses that have thickened irregular walls or septa in which enhancement can be seen.	Surgery or follow-up. Over 50% of the lesions are malignant.
IV	These lesions are clearly malignant cystic lesions that contain enhancing soft-tissue components.	Surgical therapy recommended. Mostly malignant tumor.

## Methods

Approval for this study was granted by the ethics committee of Nanjing Medical University (China). Written informed consent was obtained from the patient for publication of this report and any accompanying images.

### Patients

Data were acquired from13 patients with CRCC (10 men and threewomen) at our center from August 2004 and April 2012. The mean age was 62 years (range, 35–74 years). Four patients were symptomatic. They showed flank pain or discomfort (three cases) and indolent hematuria (one case). Nine patients were asymptomatic. Their cystic renal masses were accidentally found during health examinations. All patients received B ultrasonography, computed tomography (CT) scan, and preoperative serum creatinine determination. Glomerular filtration rate (GFR) was measured on cases preparing for partial nephrectomy. The diagnoses were made according to the Bosniak classification system.

### Surgical treatments

Patients with CRCC underwent open or laparoscopic nephrectomy. Intraoperative frozen section analysis was performed on every case. Cases with simple renal cysts received laparoscopic renal cyst decortications.

## Results

**Figure 1:**
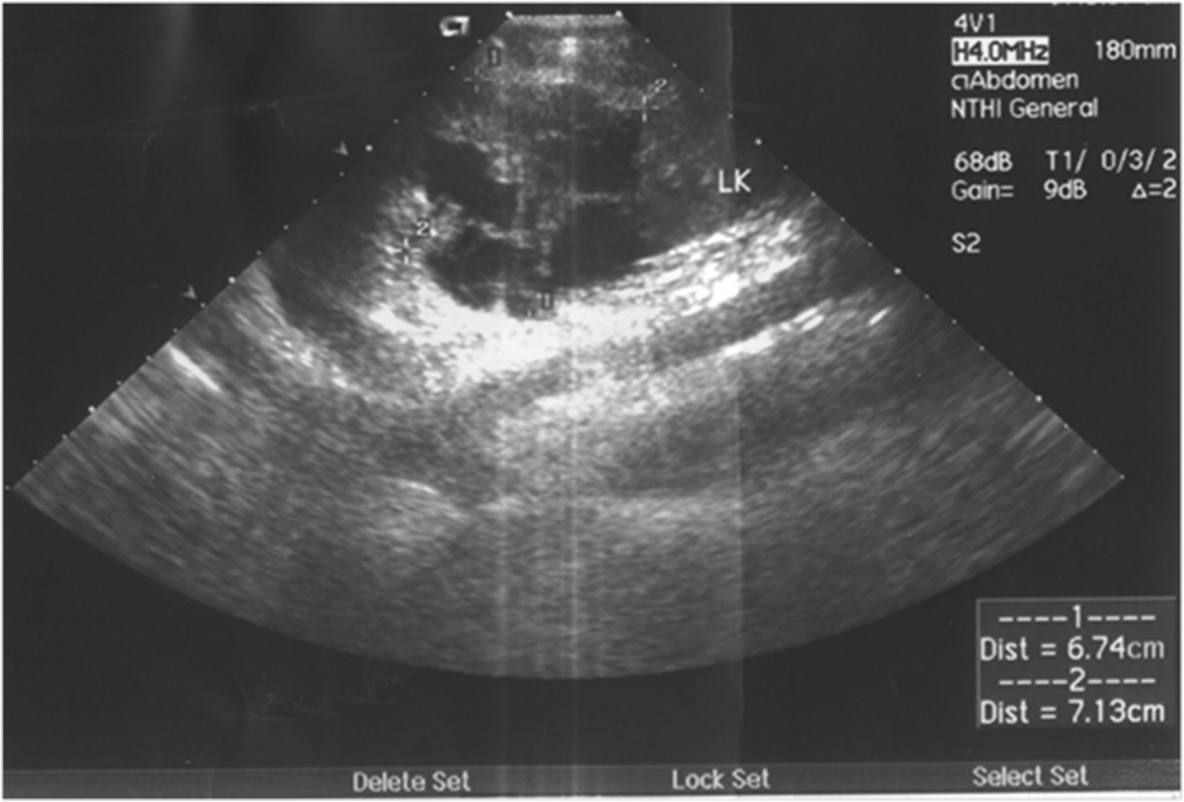
**A typical B ultasonographic image of CRCC.** The uneven cystic walls, hyperechoicsepa, and nodules were visible.

**Figure 2:**
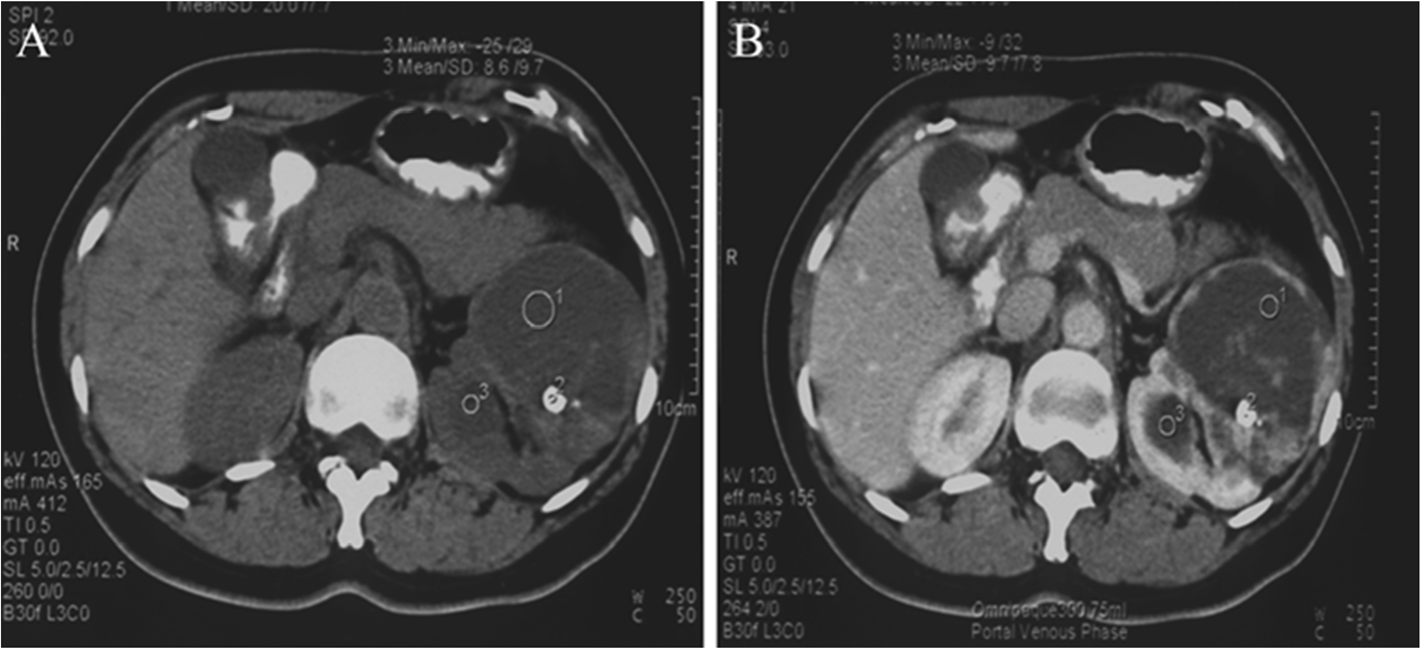
**A typical CT scan image of a left kidney with CRCC.** (**A**)The CT scan showed the thick and irregular capsule walls surrounding several cysts with hyperdense septa and nodules. (**B**)The enhanced CT scan image showed the intense enhancements and calcification of capsule walls, septa, and nodules. The debris, floc, and blood clots were also visible in the hydatid fluid.

The preoperative serum creatinine concentration and GFR were in normal range. Left renal cystic masses were observed in eight patients. The remaining five showed right renal cystic masses. Of the 13 patients, ninemultilocularCRCC, one unilocular CRCC, and three simple renal cysts were diagnosed using B ultrasonography (Figure 1). After CT scanning (Figure 2), one ‘simple renal cyst’ was diagnosed as unilocularCRCC. The mean diameter of the masses was 6.2 cm (range, 3.6-8.5 cm). No tumor metastasis was observed through preoperative examinations. According to the TNM staging system, all patients were in the cT_1-2_N_0_M_0_ stage.

The 11 patients diagnosed with CRCC underwent nephrectomy, with one case of open radical nephrectomy, six of laparoscopic radical nephrectomy, and four of open partial nephrectomy. Intraoperative frozen section analysis certified to preoperative diagnosis of CRCC. The two patients diagnosed with simple renal cysts received laparoscopic cyst decortication. During the operation, dark red hydatid fluid and abnormal capsule walls were observed in one case. Intraoperative frozen section analysis of excised capsule walls suggested renal clear cell carcinoma. The procedure was then changed to laparoscopic radical nephrectomy. Postoperative pathology confirmed the diagnosis of CRCC (clear cell carcinoma). The final pathological result confirmed the diagnosis of clear cell carcinoma, although no abnormalities were observed in another patient during operation. The patient was readmitted for open radical nephrectomy 1 month later.

After operation, all patients had an uneventful recovery. During the follow-up (range, 6–60 months), the serum creatinine concentrations and GFR of the partially removed kidneys remained stable within the normal range. No tumor recurrence or metastasis occurred.

## Discussion

Cystic degeneration of the kidney is very common in renal lesions. About 50% of individuals aged >50 years have cystic renal disease. However, CRCC is relatively rare [3]. Accurate diagnosis and treatment are sometimes difficult because CRCC and benign renal cystic disease have similar clinical manifestations and imaging characteristics. Here, we retrospectively analyzed 13 patients with CRCC at our center and summarize the characteristics of CRCC according to available literature. The information may improve the diagnosis and treatment of this kind of disease.

### Etiology and pathologic features of CRCC

The pathogenesis of CRCC remains unclear. Some possible reasons are as follows. First, the tumor originates from the epithelium of the proximal convoluted tubule and grows in a cystic pattern. Then the cystic neoplasm gradually forms mutually unintelligible cysts with different sizes, containing hemorrhages and pseudo-capsules. This kind of tumor is usually multilocularCRCC. Second, insufficient blood supply, hemorrhage, and necrosis of renal cell carcinoma can lead to the formation of pseudocysts. This kind of tumor is usually unilocularCRCC and shows thick and irregular capsule walls. Third, the tumor originates from the epithelial cells of the cyst wall. The mass is usually located in the base of the cyst. Lastly, the tumor may obstruct the kidney tubules and renal arterioles, which can cause cysts to form. This kind of CRCC is relatively rare. In the present study, nine cases were found to be multilocular and four were unilocular.

Pathologically, the mass of CRCC usually has clear boundaries and is composed of capsular cavities of different sizes [4]. At the level of electron microscopy, clear cancer cells and scar-like tissue can be observed. Cancer cells are uniform in size and rare in mitotic figures, with low nuclear grade (diploid or few aneuploidy) [4,5]. Cyst puncture cytology examination can assist diagnosis through observing abnormal hydatid fluid and cells. However, the low positive rate limits its use in clinical settings. Intraoperative pathological examination is more accurate for diagnosis. In our study, frozen section analysis not only confirmed the preoperative imaging diagnosis of 11 cases with CRCC, but also prevented misdiagnosis in one case. Our data suggest that pathological examination is necessary for the diagnosis and further treatment of CRCC, especially suspected cases.

### Clinical manifestation of CRCC

The triad of hematuria, flank pain, and abdominal mass is the classic symptoms of renal cell carcinoma, including CRCC. However, only 10% of patients with renal cell carcinoma have all three signs. This often means that tumor is in the advanced stage. The majority of patients with CRCC are identified despite the absence of clinical symptoms. In our study, nine asymptomatic patients with CRCC were identified through routine physical examinations. Only four patients showed typical symptoms.

### Imaging characteristics of CRCC

B ultrasonography and CT scan are the main methods of diagnosing CRCC [6,7]. Typical ultrasound images are as follows: cystic echo-free masses with hyperechoic septa, thick capsule walls, and septa with hyperecho, and several hyperechoic nodules attached to these septa. CT scan can provide richer diagnostic information than ultrasonography. It can show the thick and irregular capsule walls surrounding the cysts with hyperdense septa and nodules. In enhanced CT scan images, the capsule walls, septa, and nodules show intense early enhancement. In addition, coarse and crescent calcification is often observed in the capsule walls, septa, and nodules. The septa tend to be of uneven thickness (often >1 mm in diameter) and nodular thickening can appear at the junctions to the capsule walls. The hydatid fluid contains debris, floc, and blood clots, which appear uneven on CT scan. The lesions have unclear borders adjacent to renal parenchyma. According to imaging features, most CRCC can be diagnosed accurately. However, a few CRCC, especially unilocularCRCC, have characteristics similar to those of simple renal cysts. In the present study, two cases were misdiagnosed as renal cysts.

### Treatment of CRCC

Nephrectomy is the most effective treatment for CRCC [8]. Corica*et al*. compared several surgical approaches of CRCC, including radical nephrectomy, simple nephrectomy, partial nephrectomy, and tumor enucleation [9]. Their results showed the best approaches to be radical and partial nephrectomy. It is now generally recognized that localized renal cancers are best treated by nephron-sparing surgery (partial nephrectomy). As long as the resection margin of the tumor is negative, partial nephrectomy is sufficient to avoid local recurrence. Nephron-sparing surgery is particularly suitable for patients with contralateral renal insufficiency or solitary kidneys. Recently, some new concepts and techniques regarding partial nephrectomy have been used to maximize the protection of renal function. In a series of studies, Simone *et al*. reported zero-ischemia combination therapy of superselective arterial embolization with partial nephrectomy to T_1_ renal carcinomas without hilar clamping [10,11]. In a recent study, they also described the use of sutureless partial nephrectomy in the treatment of small and exophytic renal carcinomas without clamping hilar vessels and reconstructing the renal parenchyma [12]. Gill *et al*. reported a novel approach to facilitate zero-ischemia partial nephrectomy without hilar clamping, in which tumor-specific or higher-order renal arterial branches were microdissected and blocked using neurosurgical aneurysm micro-bulldog clamps [13,14]. Intraparenchymal renal carcinomas could also be treated by partial nephrectomy with the help of intraoperative ultrasound guidance [15]. These developments reduced ischemic renal damage, protected renal function, and expanded the indications for partial nephrectomy. However, because of the complexity of the procedure involved, nephron-sparing surgery should be performed at well-equipped centers by experienced personnel. Partial nephrectomy began to be widely performed for renal carcinoma at our center since 2008. In the present study, most of the nine patients undergoing radical nephrectomy were admitted before 2008 and had large renal mass. The four cases receiving partial nephrectomy were admitted after 2008 and had T_1_ stage. Open nephrectomy was the gold standard treatment for CRCC. Recently, laparoscopic nephrectomy and even robot-assisted nephrectomy have become more widely used [13,14]. With the development of our experience and skills, laparoscopic partial nephrectomy has now been the first-line therapy for renal carcinoma at our center if indications are appropriate.

According to our experience, active surveillance or aspiration biopsy should be performed on suspected cystic renal masses <3 cm in diameter. CRCC should be considered if the capsule walls thicken rapidly or if the contents of the cyst change. If the size of the cystic renal lesions is >3 cm or if the imaging characteristics do not meet the performance of renal cysts, surgical exploration is needed. Intraoperative pathological examination may facilitate accurate diagnosis and help clinicians develop further surgical approaches. However, a few cases with CRCC do not show any malignant signs. In our study, one ‘simple renal cyst’ case was diagnosed as CRCC and the patient underwent open radical nephrectomy 1 month later. For patients unsuitable for surgery, renal artery embolism, radiofrequency ablation, immunotherapy, molecular target therapy, and radiotherapy can be considered. However, the exact effect remains controversial.

### Prognosis of CRCC

CRCC has better prognosis due to low nuclear grade and TNM stage, regardless of tumor size [16]. The 10-year survival rates and non-recurrence rate after operation were 97.3% and 90.3%, respectively [17]. Although there are no specific guidelines, a follow-up every 6 months during the initial 3 years and then every 1 year is suitable.

In the present study, we retrospectively analyzed a series of patients with CRCC and summarize the pathologic features, clinical manifestation, imaging characteristics, surgical treatment, and prognosis of CRCC. Our study showed that most cases of CRCC can be diagnosed and treated promptly and accurately through the combination of imaging examinations (B ultrasonography and CT scan) and intraoperative pathological examination. Nephrectomy is the first-line therapy. Nephron-sparing surgery should be preferred for CRCC. The prognosis of CRCC is good after a successful operation.

## Conclusions

CRCC is a special type of renal cell carcinoma. The diagnosis and treatment of CRCC are sometimes difficult because its clinical manifestations and imaging characteristics can be similar to those of benign renal cystic disease. By combining imaging examinations (B ultrasonography and CT scan) with intraoperative pathological examination, most cases of CRCC can be diagnosed and treated promptly and accurately. Nephrectomy is the first-line therapy. Nephron-sparing surgery should be preferred for CRCC. The prognosis of CRCC is good after a successful operation.

## Competing interests

The authors declare that they have no competing interests.

## Authors’ contributions

JZ and BL participated in the operation and wrote the paper. NS and MG designed the study and did the operation. LH and ZW participated in the operation. CY helped to do draft the manuscript. All authors read and approved the final manuscript.
